# Polylactide-Based Films Incorporated with Berberine—Physicochemical and Antibacterial Properties

**DOI:** 10.3390/foods12010091

**Published:** 2022-12-24

**Authors:** Ewa Olewnik-Kruszkowska, Magdalena Gierszewska, Magdalena Wrona, Agnieszka Richert, Anna Rudawska

**Affiliations:** 1 Department of Physical Chemistry and Physicochemistry of Polymers Faculty of Chemistry, Nicolaus Copernicus University in Toruń, Gagarin 7 Street, 87-100 Toruń, Poland; 2 Aragon Institute of Engineering Research I3A, Department of Analytical Chemistry, University of Zaragoza, Torres Quevedo Building, María de Luna St. 3, E-50018 Zaragoza, Spain; 3 Department of Genetics, Faculty of Biological and Veterinary Sciences, Nicolaus Copernicus University in Toruń, Lwowska 1 Street, 87-100 Toruń, Poland; 4 Faculty of Mechanical Engineering, Lublin University of Technology, Nadbystrzycka 36 St., 20-618 Lublin, Poland

**Keywords:** polylactide, berberine, active compounds, antibacterial properties, packaging materials

## Abstract

A series of new polymeric materials consisting of polylactide (PLA), polyethylene glycol (PEG) and berberine chloride (B) was evaluated. PEG was incorporated into the polymer matrix with the aim of obtaining a plasticizing effect, while berberine was added in order to obtain antibacterial properties in formed packaging materials. Materials were formed using the solvent-casting procedure. Fourier transform infrared spectroscopy and scanning electron microscopy were used so as to establish the structural changes resulting from the introduction of berberine. Thermogravimetry and differential scanning calorimetry were applied to study the thermal properties. Further, mechanical properties and differences in colour and transparency between the control sample and films containing berberine were also studied. The recorded data indicates that berberine formed a network on the surface of the PLA-based materials. Introduction of an active compound significantly improved thermal stability and greatly affected the Young’s modulus values of the studied polymeric films. Moreover, it should be stressed that the addition of the studied active compound leads to an improvement of the antibacterial properties, resulting in a significant decrease in growth of E. coli and the S. aureus bacteria cultures.

## 1. Introduction

Contamination with pathogenic micro-organisms is a considerable threat, especially in the food industry, where the presence of micro-organisms in the packaging in which food is stored may cause accelerated decomposition of products. Resistant micro-organisms are particularly problematic as they quickly and easily mutate their genes, thus making their elimination difficult. Until now, food spoilage was mainly inhibited by the introduction of different types of preservatives. However, the current trend to promote the consumption of food as free of additives as possible makes it necessary to find new ways of storing it [1,2].

Another problem that has to be taken into consideration is the contamination of the environment with plastic packaging. Of late, the widespread use of plastic food packaging has been identified as a risk factor to the natural environment. Therefore, currently, more focus is devoted to the development and application of biodegradable polymers that meet the requirements for so-called “green packaging”. Poly (lactic acid), chitosan, starch and cellulose belong to the most popular compounds used in the production of biodegradable packaging materials. In order to broaden the areas of possible use of packaging incorporating the materials mentioned above, it is necessary to modify these compounds by adding antibacterial agents in the industrial manufacturing process [3,4]. The application of polymeric materials with antimicrobial properties is gaining more and more interest from both the scientific and the commercial point of view.

In literature, polylactide-based antibacterial packaging films were formed by the incorporation of the following compounds: essential oils (tea tree, cinnamon, clove) [5,6,7], plant extracts such as natural coffee, cocoa or cinnamon extracts [8], polyhexamethylene guanidine derivatives [9], nanosilver [10], and nisin [11].

Flavonoids are one of the groups of such substances. Flavonoids are known as naturally occurring polyphenolic compounds characterized by a flavan nucleus, and they constitute one of the most common classes of compounds present in fruit and vegetables. When present in plants, these compounds impede ultraviolet degradation as well as pathogen proliferation. Moreover, it should be stressed that flavonoids also possess antibacterial, antiviral, and anti-inflammatory properties [12]. In our previous work, quercetin was introduced into the PLA-matrix to form antibacterial packaging materials [13]. In the present work, one of the flavonoids known as berberine was used in order to obtain polylactide-based materials with biocidal properties.

**Figure 1:**
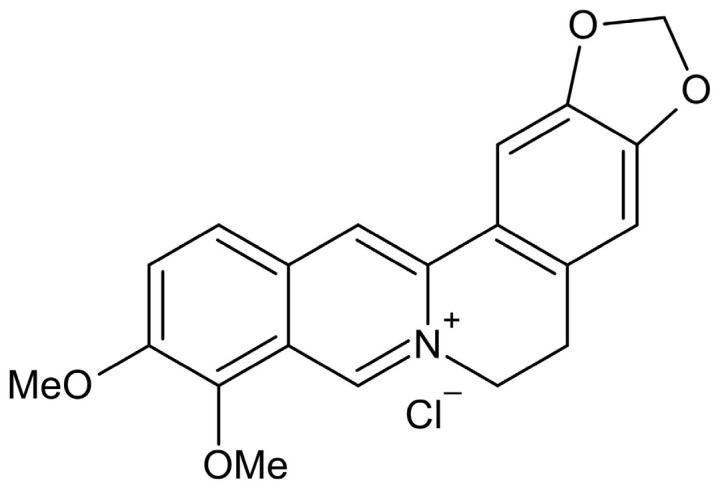
The structure of berberine.

Berberine hydrochloride, most commonly known as berberine, is an isoquinoline-derived alkaloid that is commonly found in herbal plants. It is characterized by pharmacological properties, such as anti-tumour, antibiotic and anti-inflammatory properties [14]. Moreover, it should be stressed that berberine is used as a natural medicine to treat intestinal infections by inhibiting the growth of the Helicobacter pylori bacteria. The structure of berberine is shown in Figure 1. Berberine hydrochloride is most commonly obtained by a process of extraction from herbal plants. In order to obtain a product of relatively high purity, a series of arduous steps must be performed involving dissolving, filtering, recrystallizing, as well as separating the columns. Applying these procedures is problematic and it is a significant challenge to achieve the required high-quality standard because plant extracts contain a large number of impurities. They include numerous compounds such as 2-naphthol, phenol and ferulic acid, which are very difficult to separate from raw materials using the most convenient and popular methods [15].

Due to the significant antimicrobial properties of berberine, it was decided to use it in order to ensure that the obtained material is characterized by biocidal activity. Berberine was studied as an antibacterial compound by Ma et al. [16]. In the mentioned work, berberine was modified by applying cinnamon acid and introduced into the systems consisting of gelatin, kappa-carrageenan, and glycerol. It was established that berberine significantly enhances the antimicrobial effect of the obtained materials.

Other possible applications of berberine were addressed in the work of Cometa et al. [17], where eco-friendly and cyto-compatible hydrogel formulations containing berberine and dedicated to skin treatments were developed. Formulation and evaluation of silk sericin-derived hydrogel, comprising berberine, was studied in the work of Yan et al. [18]. The obtained results indicated that the analyzed hydrogel is viable for long-term release of berberine.

This study aims to form and characterize bioactive polylactide-based materials incorporating berberine that can be used as active packaging materials. Taking into account the properties of the obtained polymeric films it seems very likely that they have great potential for application in the food industry.

## 2. Materials and Methods

### 2.1. Materials

Polylactide type 2002D was supplied by Nature Works^®^ (Minnetonka, MN, USA). The used polymer was characterized by an average molecular weight of 155,500 Da. Berberine hydrochloride and poly(ethylene glycol) with an average molecular weight of 1500 were delivered by Sigma-Aldrich (Steinheim, Germany). Chloroform and methanol were purchased from Avantor Performance Materials Poland S.A. (Gliwice, Poland).

Bacterial reference strains of Escherichia coli (ATCC 8739) and Staphylococcus aureus (ATCC 6538P) used in this study were supplied by Microbiologics^®^ (St. Cloud, MN, USA). Moreover, an agar medium consisting of tryptone peptone—15 [g/L], phyton peptone—5 [g/L], and sodium chloride—5 [g/L], was delivered by Oxoid (Hampshire, UK).

### 2.2. Polylactide-Based Materials Formation

The polylactide-based films were obtained using the following procedure. Initially, dry polylactide was dissolved in chloroform with the aim of obtaining a 3% solution. In the next stage PEG (5% *w*/*w*, PLA) and an appropriate amount of berberine (0.5%, 1%, or 2% *w*/*w*, PLA) dissolved in methanol were introduced into the solution. The resulting formulations were cast onto clean glass plates and left at ambient temperature for 48 h for solvent evaporation. The make-up of the obtained films has been presented in Table 1.

### 2.3. FTIR Analysis

The study of the impact of the introduction of berberine on the structure of the obtained films was conducted using a Nicolet iS10 (Thermo Fisher Scientific, Waltham, MA, USA). The frequency range of 500–4000 cm^−1^, and a resolution of 4 cm^−1^ were applied in all of the 64 scans during the recording of the spectra. With the aim of analyzing the obtained data, the OMNIC 7.0 software (Thermo Fisher Scientific, Waltham, MA, USA) was applied.

### 2.4. Microstructural Study

The alteration of the structure in the obtained materials due to the addition of berberine was studied by means of a scanning electron microscope (Quanta 3D FEG, FEI Company, Hillsboro, OR, USA). Images of all films dusted with a layer of gold were taken at 2500× and 15,000× magnification.

### 2.5. Thermal Properties

The thermal properties of the PLA, berberine and PEG films were studied by applying the differential scanning calorimetry and the thermogravimetry methods. In the case of DSC analyses, the following conditions were applied: the heating rate was 10 °C/min, and the temperatures ranged between 25 and 200 °C in a nitrogen atmosphere. Moreover, it should be mentioned that a thermoanalyzer produced by Polymer Laboratories (Epsom, UK) was used during all performed measurements.

Thermogravimetric (TG) analyses of the obtained films with and without the addition of berberine were performed on Simultaneous TGA-DTA Thermal Analysis type SDT 2960 (TA Instruments, London, UK). The thermal decomposition of the obtained materials was performed at a heating rate of 10 °C/min under air flow, from room temperature to 600 °C. Three repetitions were performed for every sample.

### 2.6. Mechanical Properties

The mechanical properties such as elongation at break (ε), tensile stress (σ_m_), and Young’s modulus (E) of the analyzed materials were studied using the testing Shimadzu EZ-test SX machine (Kyoto, Japan). The crosshead speed was 10 mm · min^−1^ with an applied 100 N force. It should be noticed that in the case of all materials at least seven samples were studied.

### 2.7. Transparency

The transparency of the studied materials was measured according to the method described in our previous work [19] at 600 nm (A600) using UV spectrophotometer Halo DB-20 (Dynamica Scientific Ltd., Newport Pagnell, GB). The transparency (T) of the polymeric films was calculated based on the data from ten measurements by applying the following Equation (1):

(1) $$T\;[mm^{-1}]=\frac{A_{600}}{d},$$

where d represents the film thickness [mm].

### 2.8. Colour Changes

The influence of berberine on the colour changes of the formed films was established using a MICRO-COLOR II LCM 6 (Dr. Bruno Lange GmbH & Co. KG, Berlin, Germany) colorimeter and the CIE L*a*b* system. According to the methodology presented in our previous work [20], the following parameters such as colour difference (*ΔE*), yellowness index (*YI*), and colour intensity (*C*), were calculated using the Equations (2)–(4) presented below:

(2) $$\Delta E=\sqrt{{\left(L-L^{*}\right)}^{2}+{\left(a-a^{*}\right)}^{2}+{\left(b-b^{*}\right)}^{2}},$$

(3) $$YI=\frac{142,86\;\cdot \;b}{L},$$

(4) $$C=\sqrt{{\left(a\right)}^{2}+{\left(b\right)}^{2}},$$

where *L* describes lightness, and *a* is the parameter representing the colours ranging from green to red, while *b* encompasses the parameter change from blue to yellow. For every polymeric film, three measurements were performed.

### 2.9. Assessment of Antibacterial Properties

The antibacterial capacity of the obtained films was established according to the method presented in our previous work [13]. The presence or absence of zones inhibiting the proliferation of *E. coli* and *S. aureus* was established after the end of the incubation time. In the case of the materials where bacterial propagation on the medium within the sample’s effective radius was not observed, a good antibacterial effect was recorded (according to Table 2 presented in the work [13]). For every polymeric film, three measurements were performed.

### 2.10. Statistical Assessment

The univariate analysis of variance (ANOVA) with the statistical significance set at *p* ≤ 0.05 was applied with the aim of establishing the statistically significant differences after berberine introduction. Calculations were performed by means of PS IMAGO PRO 8.0 SPSS Statistics (IBM Corporation, New York, NY, USA) program.

## 3. Results and Discussion

### 3.1. Structural Analysis

**Figure 2:**
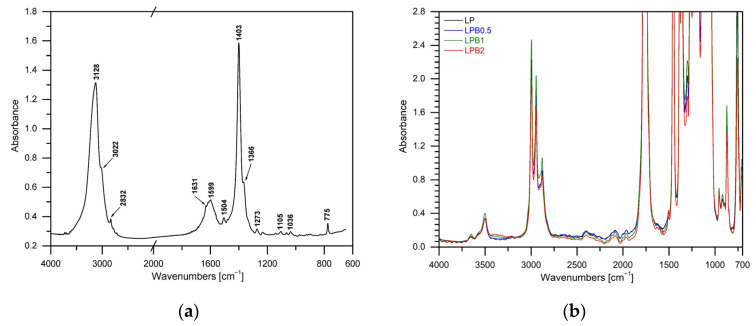
FTIR spectrum of berberine (**a**) and FTIR spectra of the PLA-based materials (**b**).

Based on the results obtained using the FTIR technique, the structure of berberine, PLA with the addition of plasticizer, as well as polymeric films containing berberine in the range 0.5–2%wt. were studied. In Figure 2a the spectrum of berberine can be noted. According to the available literature [21,22,23], characteristic groups comprising the berberine structure were indicated. The presence of phenolic O-H groups has been proved by the broad band with a maximum at 3128 cm^−1^. Moreover, in the disused spectrum of pure berberine, bands at 3022 cm^−1^, 2832 cm^−1,^ and 1403 cm^−1^ represent the symmetric and asymmetric stretching vibrations of the C-H groups. The aromatic C=C stretching vibrations correspond to the characteristic bands at 1599 and 1504 cm^−1^. Additionally iminium (C=N^+^) groups have been observed at 1631 cm^−1^. In the range between 700–1300 cm^−1^ skeletal C-C vibrations can be clearly seen.

The spectra of the studied materials have been presented in Figure 2b. The structures of pure polylactide as well as films consisting of polylactide and poly(ethylene glycol) are well-known and were described in detail in our previous publications [5,13,20,24].

However, during the analysis of the spectra of the obtained materials incorporating 0.5%wt., 1%wt. and 2%wt. of the biocidal compound, the flattening of the band with the frequency of 3126 cm^−1^ has been noted, which corresponds to the stretching vibrations, the O-H group. Moreover, it should be stressed that in the spectra of LPB0.5, LPB1 and LPB2, when compared to the foil without berberine, no new bands were recognized, only the intensity of the existing ones changed. This phenomenon can be observed especially in the range of 3500 cm^−1^ and 2998–2945 cm^−1^, where vibrations can be observed corresponding to the -OH groups, and the asymmetric and symmetrical vibrations of CH_3_ and CH, respectively.

### 3.2. SEM Results

**Figure 3:**
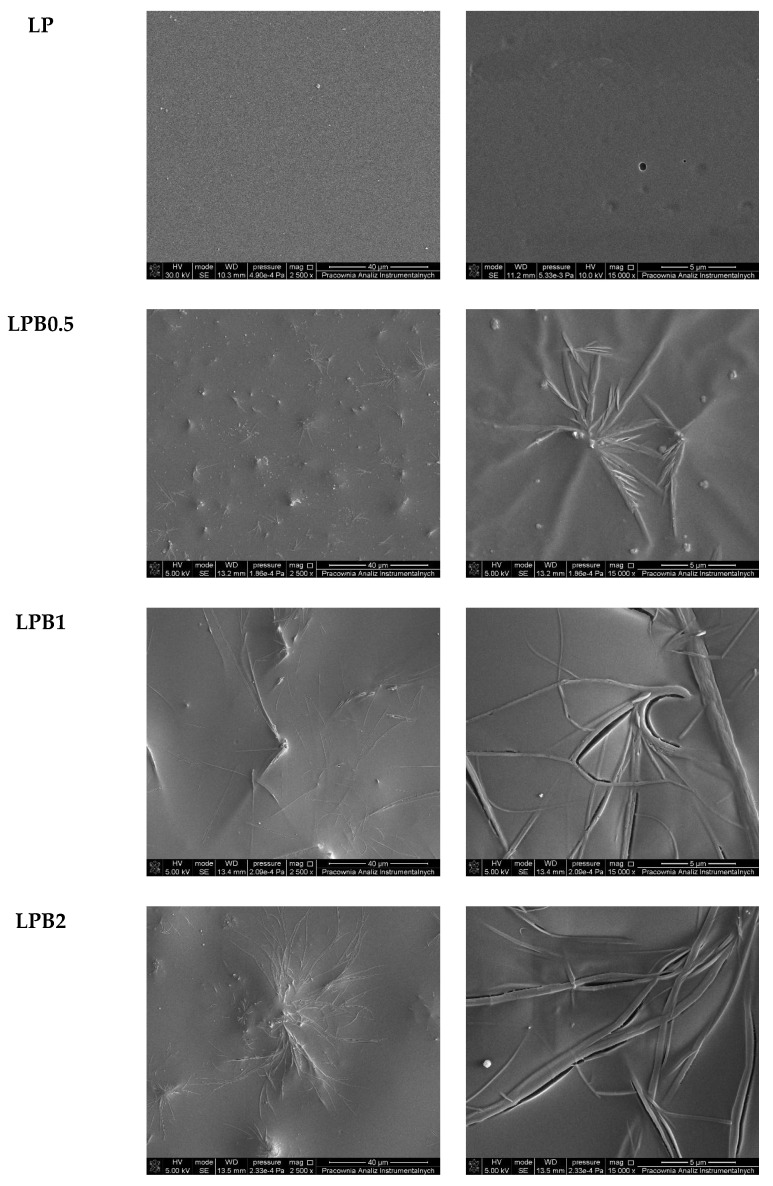
SEM images of studied materials.

In order to establish the influence of berberine on the microstructure of the formed materials, SEM technique was applied. In Figure 3 the photographs of all films with the two different magnifications (2500× and 15,000×) are shown. The film consisting of polylactide with the addition of a plasticizer (LP) was characterized by a smooth and uniform surface.

The incorporation of berberine into the LP system has a notable impact on the morphology of the obtained materials. Figure 3 depicts also the surface of the films comprising 0.5%wt., 1%wt. and 2%wt. of the biocidal agent respectively. In the case of all three of the above-mentioned samples, characteristic structures on the surface can be observed. Their presence indicates the crystallization process of berberine has taken place during the polymeric film-forming, and for this reason, the structures can be observed in the form of irregularly shaped filaments or needles embedded in the surface of the films. Moreover, the obtained results revealed that with the increasing amount of berberine in the polymeric matrix, the number and size of the scattered structures increases. This phenomenon indicates that the blends of the polymers and additives are immiscible [25].

### 3.3. Changes in Thermal Properties of Polylactide-Based Films

**Figure 4:**
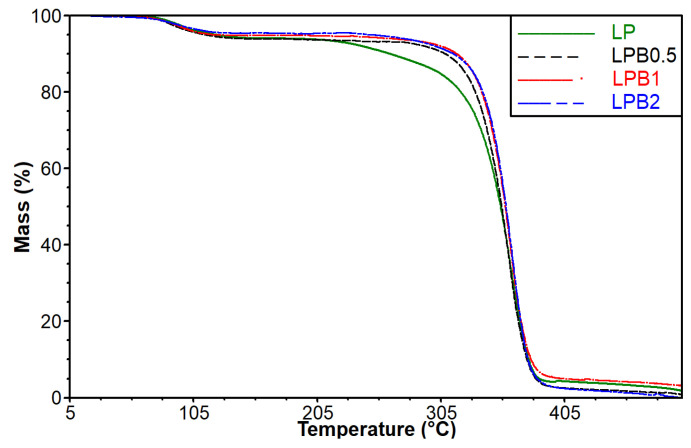
TG curves for polylactide-based materials both comprising and lacking berberine.

It is well-known that different additives can significantly influence the thermal properties of formed polymeric materials. For this reason, thermogravimetric analysis, as well as differential scanning calorimetry, were conducted. In Figure 4 the thermogravimetric curves relating to the studied materials are depicted.

With the aim of establishing the range of changes in thermal stability, the values of temperatures at 10%, 30%, and 50% mass loss of the studied polymeric materials, both comprising and lacking berberine, were taken into account. The obtained results are shown in Table 2.

**Table 2:** TG data for studied PLA-based films.

Sample	Temperature (°C) at Mass Loss		
	10%	30%	50%
LP	262.5 ± 1.2	337.5 ± 1.4	353.3 ± 2.0
LPB0.5	308.0 ± 2.2 ^A^	342.3 ± 1.0 ^A^	354.1 ± 2.1
LPB1	314.6 ± 1.1 ^A,B^	347.4 ± 0.2 ^A^	357.6 ± 1.6 ^A^
LPB2	317.4 ± 0.8 ^A,B,C^	346.4 ± 0.5 ^A,C^	357.0 ± 1.5

*p* < 0.05, statistically significant differences in comparison: ^A^—to LP film, ^B^—to LPB0.5 sample, ^C^—to LPB1 material.

The data indicate that incorporation of berberine into the PLA matrix containing 5%wt. of plasticizer leads to a rise in thermal stability of the obtained materials. It needs to be emphasized that the increase in thermal stability after the incorporation of flavonoids into the different polymeric matrices was observed in literature [13,26,27].

It has to be highlighted that the most notable changes in thermal stability were recorded in relation to the film having a 0.5%wt. content of berberine. In the case of the mentioned film (LPB0.5), the temperature values for a 10% mass loss increased by 45.5 °C in comparison to the LP sample. However, the differences between temperatures at 30% and 50% mass loss of control material (LP) and the film containing 0.5%wt. of berberine are characterized by significantly lower values. Moreover, the tests reveal that the introduction of higher amounts of berberine (1 and 2%wt.) did not affect the multiplication of thermal stability. Taking into account that pure berberine is not stable above 78 °C [28], the obtained results seem to be surprising. However, it should be stressed that in the structure of the active additive, nitrogen and chlorine atoms, which are known as flame retardants, can be observed. Presumably, during the decomposition of berberine, nitrogen and chlorine-based compounds are formed, which may significantly affect the stability of the polymer.

**Figure 5:**
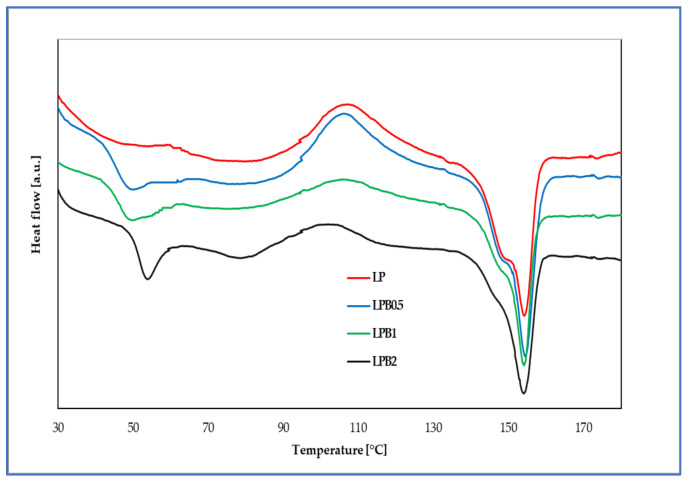
DSC curves of the analyzed polymeric materials.

In Figure 5 DSC curves for the polymeric films named LP, LPB0.5, LPB1, and LPB2 are shown.

Based on the conducted analyses the melting (T_m_), glass transition temperature (T_g_) values, and temperatures of crystallization (T_c_), as well as the enthalpies, ΔH_m_ and ΔH_c_, were determined and are presented in Table 3. Additionally, the degree of crystallinity (X_c_) was calculated and is also shown in Table 3.

Considering the recorded data, it can be seen that the introduction of berberine determines the values of the glass transition temperature, which indicate that below the T_g_ values, the macromolecules can move in relation to each other for short distances and materials remain in the glassy state. Furthermore, molecular mobility below T_g_ is low, leading to higher stability and restricted movement of molecules [29]. Moreover, the addition of flavonoid influences the PLA crystallization, and therefore a decrease in the T_c_ and ΔH_c_ of the obtained materials can be observed [13]. In summary, it can be assumed that the network of berberine, observed in SEM photos, presumably is able to limit chain displacement and as a result increases T_g_ value and simultaneously decreases ΔH_c_ and T_c_.

The recorded result, however, showed that the incorporation of berberine into the LP matrix does not influence the value of melting temperature. It should be noted that the recorded results suggest a dependence between the amount of active additive and the degree of crystallinity of formed films. In the case of the LPB0.5, LPB1, and LPB2 samples the X_c_ significantly increases with the increase in berberine quantity. The melting temperature depends mainly on the molecular mass of the polymer, the size, and quality of crystallite, and the degree of crystallinity [30,31]. In the case of polymers where cold crystallization occurs, two methods of the degree of crystallinity calculation are possible. One of them takes into account the melting and cold crystallization enthalpies [32,33,34], while the other method only involves the analysis of the melting enthalpy [35]. In our study, both enthalpies were included during the calculation. Obtained data clearly indicate that berberine disrupts the formation of crystallites during the cold crystallization process. For this reason, an increase in the degree of crystallinity can be observed.

### 3.4. Changes in Mechanical Properties

**Figure 6:**
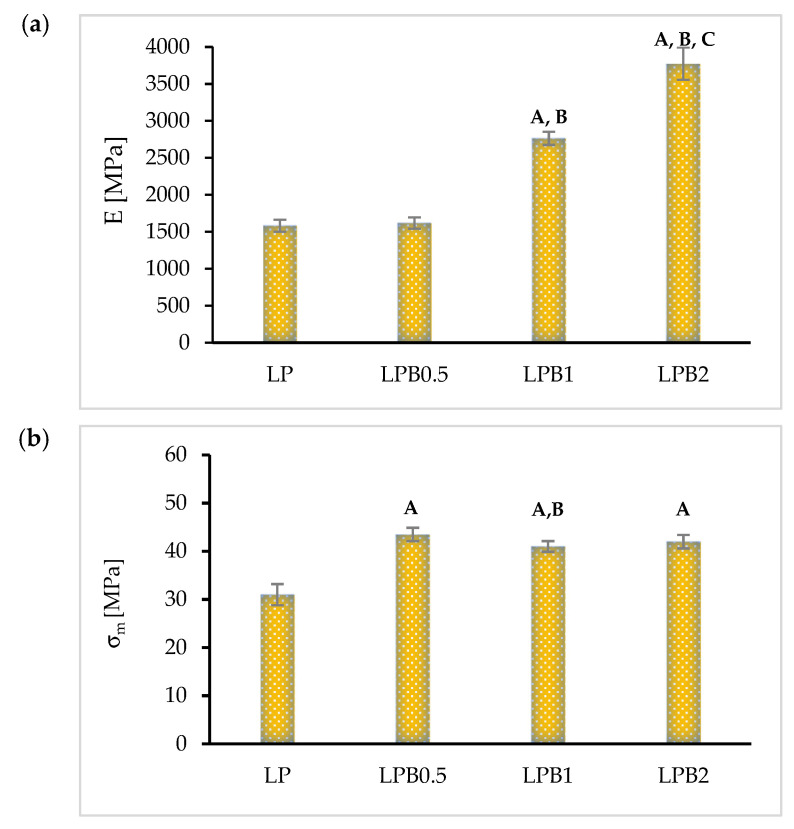

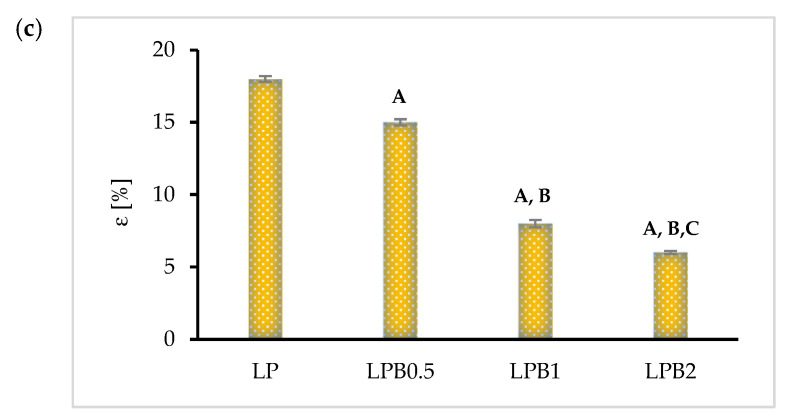
Values of mechanical properties of obtained films: (**a**) Young’s Modulus (E), (**b**) tensile strength (σ_m_), (**c**) elongation at break (ε). (*p* < 0.05, statistically significant differences in comparison: A—to LP film, B—to LPB0.5 sample, C—to LPB1 material).

Mechanical properties are among the most important factors that are taken into account when selecting a packaging material. In this paper, the attention is focused on the effect of flavonoids, such as berberine, on Young’s modulus (E), tensile strength (σ_m_), and elongation at break (ε) values presented in Figure 6a–c respectively).

It can be clearly noted that the incorporation of berberine into the polymeric matrix results in an increase of tensile strength and Young’s modulus values in comparison with an LP sample. The highest value of Young’s modulus was obtained after the introduction of 2%wt. of flavonoid into the LP system, while the values of tensile strength remained almost the same level for all films containing berberine. This can be attributed to the fact that berberine significantly changes the degree of crystallinity as well as forming a unique type of network that reinforces the structure of the obtained materials. It needs to be noted that in relation to the LPB2 sample, the Young’s modulus values were more than two times higher than in the case of the pure LP film. The aforementioned effect of berberine on the described properties has not been previously studied by other researchers.

Elongation at break (ε) values were significantly reduced after the introduction of berberine. Formation of the network mentioned above, the lower homogeneity of the matrices (shown by SEM analysis) and the increase in the degree of crystallinity gradually decreased the elongation of the polymer matrices [36]. For this reason, with the increase in the amount of active additive, the elasticity of the analyzed materials plummets. The lowest value of ε was recorded for LPB2 filled with 2.0%wt. of berberine, and equalled only about 6%. This indicates that elongation at break of the LPB2 sample is three times lower than in the case of the pure LP material.

The obtained results are in opposition to the properties of materials where quercetin (a different flavonoid) was introduced into PLA-PEG systems [13]. It can be assumed that ε values can be correlated with the structure of the used flavonoids. Luzi et al. [37] found that changes in mechanical properties were a result of inter-molecular interactions between the polyhydroxyl groups of quercetin and the hydrophilic groups of the polymer matrix. The structure of berberine is devoid of the mentioned polyhydroxyl groups and this may be one of the reasons for the reduction of elongation of break.

### 3.5. Changes in Colour and Transparency of Studied Materials

**Figure 7:**
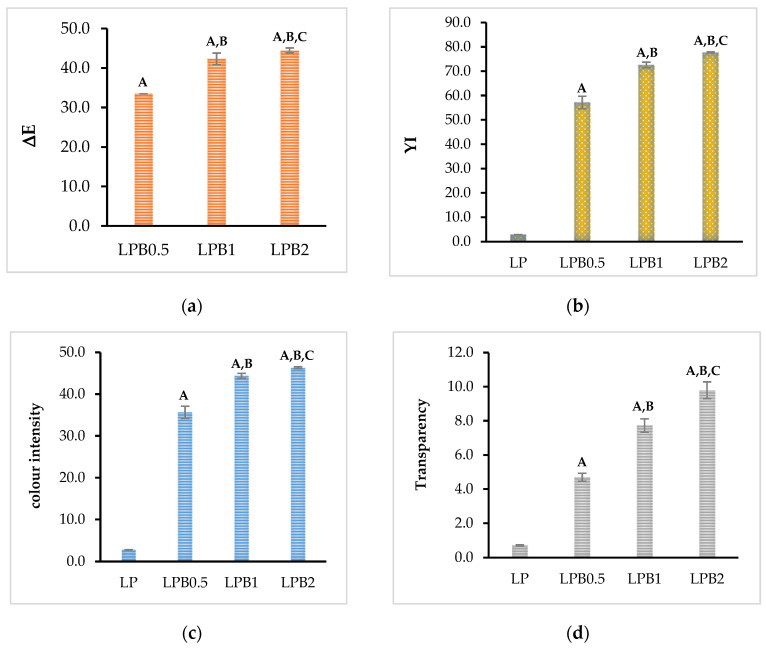
Colour parameters (**a**) ΔE, (**b**) YI, (**c**) colour intensity, and (**d**) transparency of studied polymeric films (*p* < 0.05, indicates statistically significant differences in comparison: A—to LP film, B—to LPB0.5 sample, C—to LPB1 material).

In the presented work the influence of berberine on parameters describing changes in colour was studied. Based on the obtained data L, a, and b the colour parameters such as total colour difference (*ΔE*), colour intensity (*C*), and yellowness index (*YI*) were calculated and are depicted in Figure 7. It can be clearly observed that berberine contributes to a material’s colour change. In most publications [38,39] we find that when *ΔE* achieves values above 5 the observers are able to recognize differences in the colour of the analyzed materials. According to the results presented in Figure 7 the introduction of berberine significantly modified the colour of the formed films. The most visible changes are caused by the addition of 0.5%wt. of flavonoid, where the colour difference value is above 30. The incorporation of 1%wt. and 2%wt. of berberine leads to the increase of *ΔE*; the colour modification does not, however, present a linear dependency between the amount of the added active compound and the value of the discussed parameter. The introduction of berberine results also in noticeable changes in the yellowness index (Figure 7b). The highest value of *YI* was obtained in the case of the sample filled with 2%wt. of the flavonoid. This is strongly related to the yellow colour of the antibacterial agent. It should be mentioned, however, that the yellow colour stimulates appetite and for this reason a higher yellowness index can likely constitute a desired effect. In Figure 7c the effect of a natural phenolic compound on saturation can be observed. The presented results indicate that the addition of berberine results in a similar effect, as can be seen in the case of colour differences and yellowness index. The same observation was made in our previous work, where a different flavonoid—quercetin—was introduced into the polylactide matrix [13].

In addition to colour changes, another important packaging parameter is transparency. In Figure 7d the values of opacity are depicted. Taking into account the presented results, a decrease in transparency can be observed. It was, moreover, established that changes in opacity are related to the amount of the active compound used in the procedure.

The study indicates that the most significant change can be noted in the case of a sample filled with 2%wt. of additive. This observation is consistent with research where a different yellow flavonoid was applied [13,27,40]. Presumably the limitation of transparency is caused by the crystallization of berberine resulting in the formation of a crystalline network in the polymeric matrix.

In summary, a decrease in transparency leads to the conclusion that obtained materials are very suitable for the storage of food susceptible to light.

### 3.6. Examination of Antibacterial Properties

There are various active packaging types on the market; among them, the following can be distinguished: absorbers—compounds that absorb various substances present in the environment enclosed in the package (e.g., oxygen, ethylene, moisture); emitters—solutions that release compounds into the packaging (e.g., CO_2_, antioxidants) and adapting substances. The latter cause desired changes in the packaged products, the insides of the packaging or the microflora present in the confines of the packaging. Berberine belongs to the natural additives that can improve the antibacterial capacity of the potential packaging materials [1,41,42,43].

Due to the reasons outlined above, the disk-diffusion method was chosen to estimate the influence of berberine on the microbiome contained in the packaging (Table 4). The results describing the biocidal properties of the film consisting of PLA with the addition of poly(ethylene glycol) were discussed in our previous work [13]. It was established that a sample without an active compound does not inhibit *S. aureus* and *E. coli* growth.

**Figure 8:**
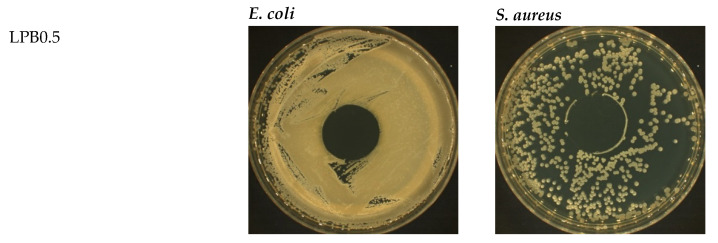

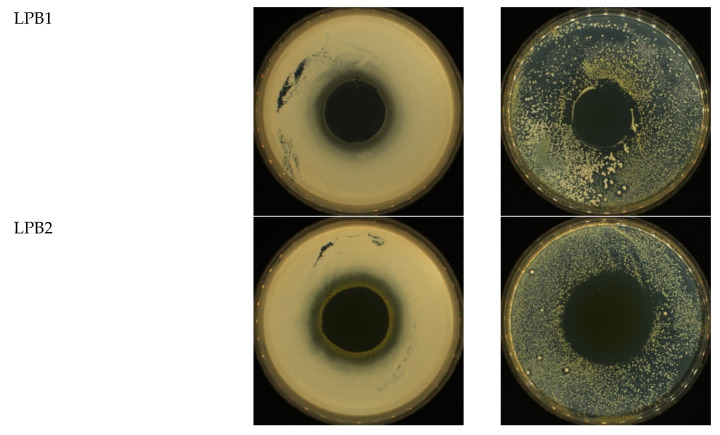
Photos of Bacteria (*E. coli* and *S. aureus*) cultures after contact with the studied polymeric films containing different amounts of berberine.

The results presented below (Figure 8) confirm that berberine significantly decreases the susceptibility of the studied materials to micro-organisms.

The incorporation of even the lowest amount of berberine, as considered in this study, leads to the formation of an antibacterial material. Moreover, it can be observed that the extent of the impediment to micro-organism proliferation is proportional to the increase in the quantity of berberine. The most significant effect of the inhibition zones of bacteria growth was observed in relation to the LPB2 film.

The antibacterial properties of berberine are well-known and described in the literature [15,21,44,45,46,47,48,49,50,51]. In the work of Hu et al. [44] *S. aureus* was employed to evaluate the antibacterial properties of berberine. It was established that materials with an addition of the flavonoid were characterized by a strong antibacterial potential. Xia et al. [48] have studied the antimicrobial characteristics and the mechanism according to which berberine acts against the methicillin-resistant *Staphylococcus aureus*. In addition, Chu et al. [49] have considered and analyzed berberine as a medicinal compound for mitigating bacteria-induced infections. In all of the publications mentioned above, the significant antibacterial properties of berberine were proved.

## 4. Conclusions

In summary, a series of new polymeric films was successfully obtained containing berberine as an antibacterial agent. Notably, berberine introduced into the PLA-PEG solution seems to form immiscible blends, simultaneously causing a unique type of crystalline network to take shape and affecting the studied physicochemical and antibacterial properties. The major finding is that, as a consequence of berberine presence, an improvement of the thermal stability, Young’s modulus as well as tensile strength values were observed. Additionally, the analysis of colour parameters showed that berberine leads to the formation of yellow films that can induce positive reception of the packaging by potential customers. What is important, and should be noted as an innovation, is the fact that the materials consisting of polylactide, 5%wt. of poly(ethylene glycol) and berberine constitute promising materials characterized by excellent antibacterial properties, improved thermal stability, and enhanced mechanical resistance.

## Figures and Tables

**Table 1:** Make-up of investigated materials (L—polylactide; P—PEG, B—berberine).

Sample	PLA Content	Berberine Content %wt.	PEG Content %wt.
LP	100	0	5
LPB0.5	100	0.5	5
LPB1	100	1	5
LPB2	100	2	5

**Table 3:** DSC data for studied PLA-based films.

Sample	T_g_ (°C)	ΔH_c_ (J/g)	T_c_ (°C)	ΔH_m_ (J/g)	T_m_ (°C)	X_c_ [%]
LP	0	22.7	107.1	24.6	154.1	1.8
LPB0.5	45.3 ^A^	19.4 ^A^	106.0 ^A^	21.7 ^A^	154.5	2.3 ^A^
LPB1	47.5 ^A^	13.4 ^A,B^	105.4 ^A,B^	21.3 ^A^	154.2	7.7 ^A,B^
LPB2	51.5 ^A^	10.3 ^A,B,C^	102.1 ^A,B,C^	21.2 ^A^	154.1	10.7 ^A,B,C^

Indicates statistically significant differences in comparison: ^A^—to LP film, ^B^—to LPB0.5 sample, ^C^—to LPB1 material.

**Table 4:** Assessment of antibacterial properties of the studied polymeric films containing berberine.

Sample	Diameter of Inhibition Zones of Bacteria Growth [mm]		Bacteria Growth in Direct Contact with Sample		Evaluation of Antibacterial Effect ^1^	
	*S. aureus*	*E. coli*	*S. aureus*	*E. coli*	*S. aureus*	*E. coli*
LPB0.5	0 ± 0	0 ± 0	lack	lack	good	good
LPB1	2 ± 0	0 ± 0	lack	lack	good	good
LPB2	8 ± 1	2 ± 0	lack	lack	good	good

^1^ in accordance with ISO 20645:2006 standard.

## Data Availability

The data presented in this study are available on request from the corresponding author.
